# Supplementary material for: Proteotranscriptomic Profiling of the Toxic Mucus of Kulikovia alborostrata (Pilidiophora, Nemertea)
Source: Toxins (Basel). 2024 Dec 26;17(1):5. doi: 10.3390/toxins17010005 (PMC11768458; doi:10.3390/toxins17010005)
Supplement: Supplementary file 1 [file toxins-17-00005-s001.zip › Supplementary File S4.zip!/ORF 006024 LINALB/230688690e3e92c6/hit_report.pdf]

Phyre2

Email	vas9i-kz@mail.ru
Description	ORF_006024_LINALB
Date	Fri Apr 7 01:53:58 BST 2023
Unique Job ID	230688690e3e92c6

Detailed template information

#	Template	Alignment Coverage	3D Model	Confidence	% i.d.	Template Information
1	c6av8A_	Alignment	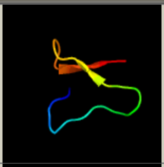	88.4	48	**PDB header:**toxin<br>**Chain:** A: **PDB Molecule:**u5-theraphotoxin-hs1b 1;<br>**PDBTitle:** exploring cystine dense peptide space to open a unique molecular2 toolbox<br>**PDB Entry:** PDBe RCSB PDBj
2	c7rz3A_	Alignment	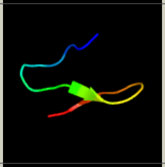	38.5	58	**PDB header:**toxin<br>**Chain:** A: **PDB Molecule:**xt3a;<br>**PDBTitle:** the solution structure of remipede double-ick toxin phi-xibalbin3-xt3a<br>**PDB Entry:** PDBe RCSB PDBj
3	c5t4rA_	Alignment	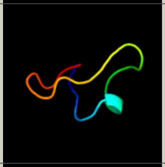	51.3	50	**PDB header:**toxin<br>**Chain:** A: **PDB Molecule:**mu-theraphotoxin-pn3a;<br>**PDBTitle:** nmr solution structure of the nav1.7 selective spider venom-derived2 peptide pn3a<br>**PDB Entry:** PDBe RCSB PDBj
4	c5we3A_	Alignment	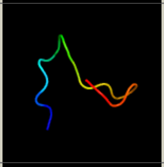	42.1	61	**PDB header:**toxin<br>**Chain:** A: **PDB Molecule:**beta-theraphotoxin-ps1a;<br>**PDBTitle:** solution nmr structure of paurtx-3<br>**PDB Entry:** PDBe RCSB PDBj
5	d1qk7a_	Alignment	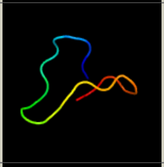	94.6	52	**Fold:** Knottins (small inhibitors, toxins, lectins)<br>**Superfamily:** omega toxin-like<br>**Family:** Spider toxins<br>**PDB entry:** PDBe RCSB PDBj
6	c6f61A_	Alignment	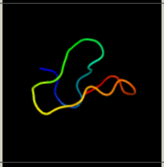	50.5	42	**PDB header:**antimicrobial protein<br>**Chain:** A: **PDB Molecule:**purotoxin-6;<br>**PDBTitle:** nmr structure of purotoxin-6<br>**PDB Entry:** PDBe RCSB PDBj
7	c7skcA_	Alignment	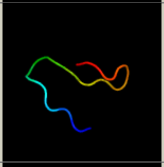	84.5	56	**PDB header:**toxin<br>**Chain:** A: **PDB Molecule:**ssp1a toxin;<br>**PDBTitle:** solution structure of spider toxin ssp1a<br>**PDB Entry:** PDBe RCSB PDBj
8	c1s6xA_	Alignment	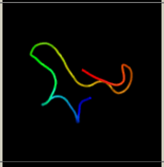	74.1	48	**PDB header:**toxin<br>**Chain:** A: **PDB Molecule:**kvap channel;<br>**PDBTitle:** solution structure of vstx<br>**PDB Entry:** PDBe RCSB PDBj
9	c1qfdA_	Alignment	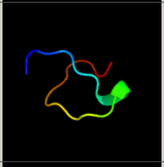	16.4	48	**PDB header:**inhibitor<br>**Chain:** A: **PDB Molecule:**protein (alpha-amylase inhibitor);<br>**PDBTitle:** nmr solution structure of alpha-amylase inhibitor (aai)<br>**PDB Entry:** PDBe RCSB PDBj
10	d1clvi_	Alignment	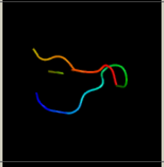	13.6	48	**Fold:** Knottins (small inhibitors, toxins, lectins)<br>**Superfamily:** Plant inhibitors of proteinases and amylases<br>**Family:** Plant inhibitors of proteinases and amylases<br>**PDB entry:** PDBe RCSB PDBj
11	c8feyA_	Alignment	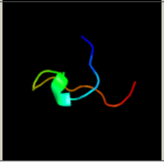	77.7	53	**PDB header:**toxin<br>**Chain:** A: **PDB Molecule:**pmu1a toxin;<br>**PDBTitle:** solution structure of pmu1a<br>**PDB Entry:** PDBe RCSB PDBj

12	d1en2a2	Alignment	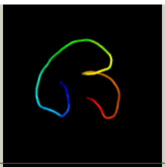	30.9	53	**Fold:** Knottins (small inhibitors, toxins, lectins)<br>**Superfamily:** Plant lectins/antimicrobial peptides<br>**Family:** Hevein-like agglutinin (lectin) domain<br>**PDB entry:** PDBe RCSB PDBj
13	d1ehda2	Alignment	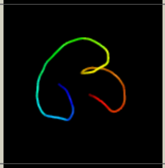	29.3	53	**Fold:** Knottins (small inhibitors, toxins, lectins)<br>**Superfamily:** Plant lectins/antimicrobial peptides<br>**Family:** Hevein-like agglutinin (lectin) domain<br>**PDB entry:** PDBe RCSB PDBj
14	c7namB_	Alignment	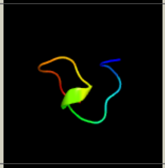	73.8	59	**PDB header:**signaling protein<br>**Chain:** B: **PDB Molecule:**trypsin inhibitor 2;<br>**PDBTitle:** lrp6_e1 in complex with lr-eet-3.5<br>**PDB Entry:** PDBe RCSB PDBj
15	c6f8eA_	Alignment	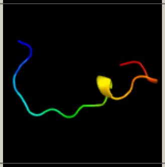	12.7	59	**PDB header:**lipid binding protein<br>**Chain:** A: **PDB Molecule:**pleckstrin homology domain;<br>**PDBTitle:** ph domain from tgaph<br>**PDB Entry:** PDBe RCSB PDBj
16	c2n86A_	Alignment	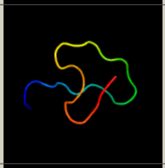	15.3	40	**PDB header:**toxin<br>**Chain:** A: **PDB Molecule:**spiderine-1a;<br>**PDBTitle:** nmr structure of ottx1a - ick<br>**PDB Entry:** PDBe RCSB PDBj
17	c1zwuA_	Alignment	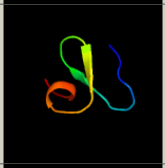	9.4	43	**PDB header:**antimicrobial protein<br>**Chain:** A: **PDB Molecule:**amaranthus caudatus antimicrobial peptide 2 (acmp2);<br>**PDBTitle:** 30 nmr structures of acamp2-like peptide with non natural beta-(2-2 naphthyl)-alanine residue.<br>**PDB Entry:** PDBe RCSB PDBj
18	c2m9lA_	Alignment	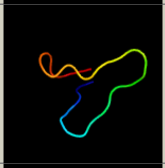	57.4	41	**PDB header:**toxin<br>**Chain:** A: **PDB Molecule:**beta-theraphotoxin-tp1a;<br>**PDBTitle:** solution structure of protoxin-1<br>**PDB Entry:** PDBe RCSB PDBj
19	c2a2vA_	Alignment	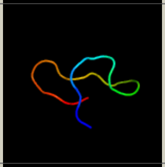	23.0	41	**PDB header:**toxin<br>**Chain:** A: **PDB Molecule:**jingzhaotoxin-xi;<br>**PDBTitle:** the solution structure of jingzhaotoxin-xi<br>**PDB Entry:** PDBe RCSB PDBj
20	c2jtbA_	Alignment	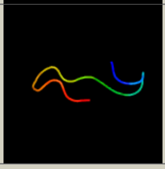	77.1	60	**PDB header:**toxin<br>**Chain:** A: **PDB Molecule:**hainantoxin-3;<br>**PDBTitle:** three dimensional solution structure of hainantoxin-iii by 2d 1h-nmr<br>**PDB Entry:** PDBe RCSB PDBj
21	c1qk6A_	Alignment	not modelled	58.7	50	**PDB header:**toxin<br>**Chain:** A: **PDB Molecule:**huwentoxin-i;<br>**PDBTitle:** solution structure of huwentoxin-i by nmr<br>**PDB Entry:** PDBe RCSB PDBj
22	c7jpmA_	Alignment	not modelled	21.7	50	**PDB header:**toxin<br>**Chain:** A: **PDB Molecule:**omega-theraphotoxin-pm1b;<br>**PDBTitle:** the solution structure of omega-theraphotoxin-pm1b isolated from king2 baboon spider<br>**PDB Entry:** PDBe RCSB PDBj
23	c6mk5A_	Alignment	not modelled	21.3	50	**PDB header:**toxin<br>**Chain:** A: **PDB Molecule:**toxin gtx1-15;<br>**PDBTitle:** solution nmr structure of spider toxin analogue [f5a,m6f,t26l,2 k28r]gptx-1<br>**PDB Entry:** PDBe RCSB PDBj
24	d1tyka_	Alignment	not modelled	9.6	50	**Fold:** Knottins (small inhibitors, toxins, lectins)<br>**Superfamily:** omega toxin-like<br>**Family:** Spider toxins<br>**PDB entry:** PDBe RCSB PDBj
25	c2wh9A_	Alignment	not modelled	90.0	64	**PDB header:**membrane protein inhibitor<br>**Chain:** A: **PDB Molecule:**guangxitoxin-1egxtx-1e;<br>**PDBTitle:** solution structure of gxtx-1e<br>**PDB Entry:** PDBe RCSB PDBj
26	d1eita_	Alignment	not modelled	34.6	56	**Fold:** Knottins (small inhibitors, toxins, lectins)<br>**Superfamily:** omega toxin-like<br>**Family:** Spider toxins<br>**PDB entry:** PDBe RCSB PDBj
27	c1eitA_	Alignment	not modelled	34.6	56	**PDB header:**neurotoxin<br>**Chain:** A: **PDB Molecule:**mu-agatoxin-i;<br>**PDBTitle:** nmr study of mu-agatoxin<br>**PDB Entry:** PDBe RCSB PDBj
28	c2aapA_	Alignment	not modelled	40.6	47	**PDB header:**toxin<br>**Chain:** A: **PDB Molecule:**jingzhaotoxin-vii;

28	c2aapA_	Alignment	not modelled	40.6	47	**PDBTitle:** solution structure of jingzhaotoxin-vii<br>**PDB Entry:** PDBe RCSB PDBj
29	c1emxA_	Alignment	not modelled	67.9	62	**PDB header:**toxin<br>**Chain:** A: **PDB Molecule:**heteropodatoxin 2;<br>**PDBTitle:** solution structure of hptx2, a toxin from heteropoda venatoria spider2 venom that blocks kv4.2 potassium channel<br>**PDB Entry:** PDBe RCSB PDBj
30	c6ceiA_	Alignment	not modelled	75.0	50	**PDB header:**toxin<br>**Chain:** A: **PDB Molecule:**gxia;<br>**PDBTitle:** solution nmr structure of conotoxin gxia from conus geographus<br>**PDB Entry:** PDBe RCSB PDBj
31	c2n3pA_	Alignment	not modelled	13.5	32	**PDB header:**toxin<br>**Chain:** A: **PDB Molecule:**asteropsin_g;<br>**PDBTitle:** solution nmr structure of asteropsin g from marine sponge asteropus<br>**PDB Entry:** PDBe RCSB PDBj
32	c5yjhA_	Alignment	not modelled	25.5	47	**PDB header:**cell adhesion<br>**Chain:** A: **PDB Molecule:**periostin;<br>**PDBTitle:** structural insights into periostin functions<br>**PDB Entry:** PDBe RCSB PDBj
33	c6f24A_	Alignment	not modelled	11.7	47	**PDB header:**lipid binding protein<br>**Chain:** A: **PDB Molecule:**c-terminal ph domain from pfaph;<br>**PDBTitle:** ph domain from pfaph<br>**PDB Entry:** PDBe RCSB PDBj
34	c5nv6A_	Alignment	not modelled	10.0	47	**PDB header:**structural protein<br>**Chain:** A: **PDB Molecule:**transforming growth factor-beta-induced protein ig-h3;<br>**PDBTitle:** structure of human transforming growth factor beta-induced protein2 (tgfbip).<br>**PDB Entry:** PDBe RCSB PDBj
35	c1kozA_	Alignment	not modelled	44.7	42	**PDB header:**toxin<br>**Chain:** A: **PDB Molecule:**voltage-dependent channel inhibitor;<br>**PDBTitle:** solution structure of omega-grammotoxin sia<br>**PDB Entry:** PDBe RCSB PDBj
36	c1mcvI_	Alignment	not modelled	22.2	57	**PDB header:**hydrolase<br>**Chain:** I: **PDB Molecule:**hei-toe i;<br>**PDBTitle:** crystal structure analysis of a hybrid squash inhibitor in2 complex with porcine pancreatic elastase<br>**PDB Entry:** PDBe RCSB PDBj
37	d1mcvi_	Alignment	not modelled	22.2	57	**Fold:** Knottins (small inhibitors, toxins, lectins)<br>**Superfamily:** Plant inhibitors of proteinases and amylases<br>**Family:** Plant inhibitors of proteinases and amylases<br>**PDB entry:** PDBe RCSB PDBj
38	d2it7a1	Alignment	not modelled	21.9	57	**Fold:** Knottins (small inhibitors, toxins, lectins)<br>**Superfamily:** Plant inhibitors of proteinases and amylases<br>**Family:** Plant inhibitors of proteinases and amylases<br>**PDB entry:** PDBe RCSB PDBj
39	c2it7A_	Alignment	not modelled	21.9	57	**PDB header:**plant protein<br>**Chain:** A: **PDB Molecule:**trypsin inhibitor 2;<br>**PDBTitle:** solution structure of the squash trypsin inhibitor eeti-ii<br>**PDB Entry:** PDBe RCSB PDBj
40	c2letA_	Alignment	not modelled	21.7	57	**PDB header:**proteinase inhibitor(trypsin)<br>**Chain:** A: **PDB Molecule:**trypsin inhibitor ii;<br>**PDBTitle:** an 1h nmr determination of the three dimensional structures of mirror2 image forms of a leu-5 variant of the trypsin inhibitor ecballium3 elaterium (eeti-ii)<br>**PDB Entry:** PDBe RCSB PDBj
41	d1h9hi_	Alignment	not modelled	20.3	57	**Fold:** Knottins (small inhibitors, toxins, lectins)<br>**Superfamily:** Plant inhibitors of proteinases and amylases<br>**Family:** Plant inhibitors of proteinases and amylases<br>**PDB entry:** PDBe RCSB PDBj
42	c6a48A_	Alignment	not modelled	13.9	42	**PDB header:**signaling protein<br>**Chain:** A: **PDB Molecule:**reelin;<br>**PDBTitle:** crystal structure of reelin n-terminal region<br>**PDB Entry:** PDBe RCSB PDBj
43	c6gftA_	Alignment	not modelled	26.3	53	**PDB header:**toxin<br>**Chain:** A: **PDB Molecule:**cyriotoxin-1a;<br>**PDBTitle:** antinociceptive evaluation of cyriotoxin-1a, the first toxin purified2 from cyriopagopus schioedtei spider venom<br>**PDB Entry:** PDBe RCSB PDBj
44	d1h9ii_	Alignment	not modelled	21.6	53	**Fold:** Knottins (small inhibitors, toxins, lectins)<br>**Superfamily:** Plant inhibitors of proteinases and amylases<br>**Family:** Plant inhibitors of proteinases and amylases<br>**PDB entry:** PDBe RCSB PDBj
45	c5i1xA_	Alignment	not modelled	59.8	44	**PDB header:**toxin<br>**Chain:** A: **PDB Molecule:**mu-trtx-pre1a toxin;<br>**PDBTitle:** resonance assignments and nmr structure determination of tarantula2 toxin, f8a mutant of beta-trtx-pre1a<br>**PDB Entry:** PDBe RCSB PDBj
46	c2mt7A_	Alignment	not modelled	56.3	44	**PDB header:**toxin<br>**Chain:** A: **PDB Molecule:**hs1a;<br>**PDBTitle:** solution structure of spider-venom peptide hs1a<br>**PDB Entry:** PDBe RCSB PDBj
47	c5o57A_	Alignment	not modelled	21.1	44	**PDB header:**signaling protein<br>**Chain:** A: **PDB Molecule:**dickkopf-related protein 4;<br>**PDBTitle:** solution structure of the n-terminal region of dkk4<br>**PDB Entry:** PDBe RCSB PDBj
48	d1d1ha_	Alignment	not modelled	16.2	36	**Fold:** Knottins (small inhibitors, toxins, lectins)<br>**Superfamily:** omega toxin-like<br>**Family:** Spider toxins<br>**PDB entry:** PDBe RCSB PDBj
49	d1la4a_	Alignment	not modelled	16.1	36	**Fold:** Knottins (small inhibitors, toxins, lectins)<br>**Superfamily:** omega toxin-like<br>**Family:** Spider toxins<br>**PDB entry:** PDBe RCSB PDBj
50	c2kusA_	Alignment	not modelled	12.2	36	**PDB header:**antimicrobial protein<br>**Chain:** A: **PDB Molecule:**sm-amp-1.1a;<br>**PDBTitle:** spatial structure of antimicrobial peptide sm-amp-1.1a<br>**PDB Entry:** PDBe RCSB PDBj
51	c2mvaA_	Alignment	not modelled	11.2	44	**PDB header:**toxin<br>**Chain:** A: **PDB Molecule:**rhtx toxin;<br>**PDBTitle:** solution structure of the toxin, rhtx

						**PDB Entry:** PDBe RCSB PDBj
52	d1nixa_	Alignment	not modelled	20.7	47	**Fold:** Knottins (small inhibitors, toxins, lectins)<br>**Superfamily:** omega toxin-like<br>**Family:** Spider toxins<br>**PDB entry:** PDBe RCSB PDBj
53	c6nk9A_	Alignment	not modelled	15.6	47	**PDB header:**toxin<br>**Chain:** A: **PDB Molecule:**aca toxin 1;<br>**PDBTitle:** solution structure of acatx1, a potassium channel inhibitor from the2 sea anemone antopleura cascaia<br>**PDB Entry:** PDBe RCSB PDBj
54	c2jyeA_	Alignment	not modelled	20.5	32	**PDB header:**cytokine<br>**Chain:** A: **PDB Molecule:**granulin a;<br>**PDBTitle:** human granulin a<br>**PDB Entry:** PDBe RCSB PDBj
55	c6v6tA_	Alignment	not modelled	14.1	32	**PDB header:**toxin<br>**Chain:** A: **PDB Molecule:**delta-theraphotoxin-hm1b;<br>**PDBTitle:** solution structure of delta-theraphotoxin-hm1b from heteroscodra2 maculata<br>**PDB Entry:** PDBe RCSB PDBj
56	c6btvA_	Alignment	not modelled	69.8	39	**PDB header:**toxin<br>**Chain:** A: **PDB Molecule:**beta-theraphotoxin-cm1b;<br>**PDBTitle:** solution nmr structures for ccotx-ii<br>**PDB Entry:** PDBe RCSB PDBj
57	c5epmC_	Alignment	not modelled	41.7	39	**PDB header:**toxin/immune system<br>**Chain:** C: **PDB Molecule:**beta-theraphotoxin-cm1a;<br>**PDBTitle:** ceratotoxin variant in complex with specific antibody fab fragment<br>**PDB Entry:** PDBe RCSB PDBj
58	c2i1tA_	Alignment	not modelled	37.9	50	**PDB header:**toxin<br>**Chain:** A: **PDB Molecule:**jingzhaotoxin-3;<br>**PDBTitle:** solution structure of jingzhaotoxin-iii, a novel toxin inhibiting both2 nav and kv channels<br>**PDB Entry:** PDBe RCSB PDBj
59	c5ki9A_	Alignment	not modelled	31.1	70	**PDB header:**antimicrobial protein<br>**Chain:** A: **PDB Molecule:**beta-defensin 104;<br>**PDBTitle:** crystal structure of human beta-defensin 4 (hbd4)<br>**PDB Entry:** PDBe RCSB PDBj
60	c6lnrA_	Alignment	not modelled	15.4	50	**PDB header:**hydrolase<br>**Chain:** A: **PDB Molecule:**class-1 chitinase;<br>**PDBTitle:** structure of intact chitinase with hevein domain from the plant2 simarouba glauca, known for its traditional anti-inflammatory3 efficacy<br>**PDB Entry:** PDBe RCSB PDBj
61	c2ys0A_	Alignment	not modelled	10.3	50	**PDB header:**hydrolase<br>**Chain:** A: **PDB Molecule:**ectonucleotide pyrophosphatase/phosphodiesterase family<br>**PDBTitle:** solution structure of the somatomedin b domain of human ectonucleotide2 pyrophosphatase/phosphodiesterase family member<br>**PDB Entry:** PDBe RCSB PDBj
62	c6ji7A_	Alignment	not modelled	55.8	58	**PDB header:**plant protein<br>**Chain:** A: **PDB Molecule:**coffeetide;<br>**PDBTitle:** coffeetides: iron-binding cysteine rich peptides from coffee waste<br>**PDB Entry:** PDBe RCSB PDBj
63	c1omgA_	Alignment	not modelled	15.3	58	**PDB header:**presynaptic neurotoxin<br>**Chain:** A: **PDB Molecule:**omega-conotoxin mviia;<br>**PDBTitle:** nmr study of omega-conotoxin mviia<br>**PDB Entry:** PDBe RCSB PDBj
64	c1dw4A_	Alignment	not modelled	15.3	58	**PDB header:**toxin<br>**Chain:** A: **PDB Molecule:**omega-conotoxin mviia;<br>**PDBTitle:** nmr structure of omega-conotoxin mviia: constraints on disulphide2 bridges<br>**PDB Entry:** PDBe RCSB PDBj
65	c7mixB_	Alignment	not modelled	15.3	58	**PDB header:**transport protein/toxin<br>**Chain:** B: **PDB Molecule:**omega-conotoxin mviia;<br>**PDBTitle:** human n-type voltage-gated calcium channel cav2.2 in the presence of2 ziconotide at 3.0 angstrom resolution<br>**PDB Entry:** PDBe RCSB PDBj
66	c1tt3A_	Alignment	not modelled	15.2	58	**PDB header:**toxin<br>**Chain:** A: **PDB Molecule:**omega-conotoxin mviia;<br>**PDBTitle:** nmr soulution structure of omega-conotoxin [k10]mviia<br>**PDB Entry:** PDBe RCSB PDBj
67	c1mviA_	Alignment	not modelled	15.1	58	**PDB header:**neurotoxin<br>**Chain:** A: **PDB Molecule:**mviia;<br>**PDBTitle:** n-type calcium channel blocker, omega-conotoxin mviia, nmr, 152 structures<br>**PDB Entry:** PDBe RCSB PDBj
68	c1dw5A_	Alignment	not modelled	15.1	58	**PDB header:**toxin<br>**Chain:** A: **PDB Molecule:**omega-conotoxin mviia;<br>**PDBTitle:** nmr structure of omega-conotoxin mviia: no constraints on disulphide2 bridges<br>**PDB Entry:** PDBe RCSB PDBj
69	c7vfuE_	Alignment	not modelled	15.0	58	**PDB header:**membrane protein<br>**Chain:** E: **PDB Molecule:**omega-conotoxin mviia;<br>**PDBTitle:** human n-type voltage gated calcium channel cav2.2-alpha2/delta1-beta12 complex, bound to ziconotide<br>**PDB Entry:** PDBe RCSB PDBj
70	c1ttkA_	Alignment	not modelled	14.9	58	**PDB header:**toxin<br>**Chain:** A: **PDB Molecule:**omega-conotoxin mviia;<br>**PDBTitle:** nmr solution structure of omega-conotoxin mviia, a n-type calcium2 channel blocker<br>**PDB Entry:** PDBe RCSB PDBj
71	c1feoA_	Alignment	not modelled	14.1	58	**PDB header:**toxin<br>**Chain:** A: **PDB Molecule:**omega-conotoxin mviia-gly;<br>**PDBTitle:** solution structure of omega-conotoxin mviia with c-terminal gly<br>**PDB Entry:** PDBe RCSB PDBj
72	c3s8vX_	Alignment	not modelled	11.8	38	**PDB header:**signaling protein<br>**Chain:** X: **PDB Molecule:**dickkopf-related protein 1;<br>**PDBTitle:** crystal structure of lrp6-dkk1 complex<br>**PDB Entry:** PDBe RCSB PDBj
						**Fold:** Knottins (small inhibitors, toxins, lectins)

73	d1hp3a_	Alignment	not modelled	10.6	55	**Superfamily:** omega toxin-like<br>**Family:** Spider toxins<br>**PDB entry:** PDBe RCSB PDBj
74	c1hp3A_	Alignment	not modelled	10.6	55	**PDB header:**toxin<br>**Chain:** A: **PDB Molecule:**omega-atracotoxin-hv2a;<br>**PDBTitle:** c-terminal truncation of omega-atracotoxin-hv2a (ct-hv2a)<br>**PDB Entry:** PDBe RCSB PDBj
75	c2dcwA_	Alignment	not modelled	9.9	55	**PDB header:**antimicrobial protein<br>**Chain:** A: **PDB Molecule:**tachystatin-b2;<br>**PDBTitle:** the solution structure of horseshoe crab antimicrobial peptide2 tachystatin b with the inhibitory cystine-knot motif<br>**PDB Entry:** PDBe RCSB PDBj
76	c2dcvA_	Alignment	not modelled	9.8	55	**PDB header:**antimicrobial protein<br>**Chain:** A: **PDB Molecule:**tachystatin-b1;<br>**PDBTitle:** the solution structure of horseshoe crab antimicrobial peptide2 tachystatin b with the inhibitory cystine-knot motif<br>**PDB Entry:** PDBe RCSB PDBj
77	c1eyoA_	Alignment	not modelled	10.7	67	**PDB header:**toxin<br>**Chain:** A: **PDB Molecule:**conotoxin tviia;<br>**PDBTitle:** solution structure of conotoxin tviia from conus tulipa<br>**PDB Entry:** PDBe RCSB PDBj
78	c1v91A_	Alignment	not modelled	13.4	75	**PDB header:**toxin<br>**Chain:** A: **PDB Molecule:**delta-palutoxin it2;<br>**PDBTitle:** solution structure of insectidal toxin delta-paluit2-nh2<br>**PDB Entry:** PDBe RCSB PDBj
79	c6xyiA_	Alignment	not modelled	10.0	60	**PDB header:**toxin<br>**Chain:** A: **PDB Molecule:**ams9.3.1;<br>**PDBTitle:** nmr solution structure of alpha-anmtx- ms11a-3 (ms11a-3)<br>**PDB Entry:** PDBe RCSB PDBj
80	c6xyhA_	Alignment	not modelled	9.9	60	**PDB header:**toxin<br>**Chain:** A: **PDB Molecule:**ams3;<br>**PDBTitle:** nmr solution structure of alpha-anmtx-ms11a-2 (ms11a-2)<br>**PDB Entry:** PDBe RCSB PDBj
81	d1e4ra_	Alignment	not modelled	9.6	50	**Fold:** Defensin-like<br>**Superfamily:** Defensin-like<br>**Family:** Defensin<br>**PDB entry:** PDBe RCSB PDBj
82	c7lyuA_	Alignment	not modelled	15.9	35	**PDB header:**signaling protein<br>**Chain:** A: **PDB Molecule:**reelin;<br>**PDBTitle:** reelin repeat 8<br>**PDB Entry:** PDBe RCSB PDBj
83	c2kraA_	Alignment	not modelled	11.9	35	**PDB header:**contractile protein<br>**Chain:** A: **PDB Molecule:**prokineticin bv8;<br>**PDBTitle:** solution structure of bv8<br>**PDB Entry:** PDBe RCSB PDBj
84	c1imtA_	Alignment	not modelled	10.9	35	**PDB header:**toxin<br>**Chain:** A: **PDB Molecule:**intestinal toxin 1;<br>**PDBTitle:** mamba intestinal toxin 1, nmr, 39 structures<br>**PDB Entry:** PDBe RCSB PDBj
85	d1ayja_	Alignment	not modelled	12.8	56	**Fold:** Knottins (small inhibitors, toxins, lectins)<br>**Superfamily:** Scorpion toxin-like<br>**Family:** Plant defensins<br>**PDB entry:** PDBe RCSB PDBj
86	c1fu3A_	Alignment	not modelled	9.7	63	**PDB header:**metal transport inhibitor<br>**Chain:** A: **PDB Molecule:**delta-conotoxin txvia;<br>**PDBTitle:** three-dimensional structure in solution of the sodium channel2 agonist/antagonist delta-conotoxin txvia<br>**PDB Entry:** PDBe RCSB PDBj
87	d1fu3a_	Alignment	not modelled	9.7	63	**Fold:** Knottins (small inhibitors, toxins, lectins)<br>**Superfamily:** omega toxin-like<br>**Family:** Conotoxin<br>**PDB entry:** PDBe RCSB PDBj
88	c5znuA_	Alignment	not modelled	16.8	83	**PDB header:**toxin<br>**Chain:** A: **PDB Molecule:**omega-conotoxin-like bu8;<br>**PDBTitle:** structure of omega conotoxin bu8<br>**PDB Entry:** PDBe RCSB PDBj
89	c1mvjA_	Alignment	not modelled	16.2	83	**PDB header:**neurotoxin<br>**Chain:** A: **PDB Molecule:**svib;<br>**PDBTitle:** n-type calcium channel blocker, omega-conotoxin mviia nmr, 152 structures<br>**PDB Entry:** PDBe RCSB PDBj
90	c1cnnA_	Alignment	not modelled	16.0	83	**PDB header:**toxin<br>**Chain:** A: **PDB Molecule:**omega-conotoxin mviic;<br>**PDBTitle:** omega-conotoxin mviic from conus magus<br>**PDB Entry:** PDBe RCSB PDBj
91	c1omnA_	Alignment	not modelled	15.7	83	**PDB header:**neurotoxin<br>**Chain:** A: **PDB Molecule:**omega-conotoxin m vii c (m seven c);<br>**PDBTitle:** solution structure of omega-conotoxin mviic, a high affinity of p-type2 calcium channels, using 1h nmr spectroscopy and complete relaxation3 matrix analysis<br>**PDB Entry:** PDBe RCSB PDBj
92	c3q8jA_	Alignment	not modelled	14.5	83	**PDB header:**toxin<br>**Chain:** A: **PDB Molecule:**asteropsin a;<br>**PDBTitle:** crystal structure of asteropsin a from marine sponge asteropus sp.<br>**PDB Entry:** PDBe RCSB PDBj
93	c1v4qA_	Alignment	not modelled	13.4	83	**PDB header:**toxin<br>**Chain:** A: **PDB Molecule:**omega-conotoxin mviic;<br>**PDBTitle:** three-dimensional solution structure of the analogue peptide of omega-2 conotoxin mviic<br>**PDB Entry:** PDBe RCSB PDBj
94	c2n7fA_	Alignment	not modelled	20.6	71	**PDB header:**toxin<br>**Chain:** A: **PDB Molecule:**muo-conotoxin mfvia;<br>**PDBTitle:** nmr solution structure of muo-conotoxin mfvia<br>**PDB Entry:** PDBe RCSB PDBj
95	c1rmkA_	Alignment	not modelled	19.7	71	**PDB header:**toxin<br>**Chain:** A: **PDB Molecule:**mu-o-conotoxin mrvib;<br>**PDBTitle:** solution structure of conotoxin mrvib<br>**PDB Entry:** PDBe RCSB PDBj
						**Fold:** Knottins (small inhibitors, toxins, lectins)

96	d1rmka_	Alignment	not modelled	19.7	71	**Superfamily:** omega toxin-like<br>**Family:** Conotoxin<br>**PDB entry:** PDBe RCSB PDBj
97	d3c8ya1	Alignment	not modelled	16.1	71	**Fold:** Fe-only hydrogenase<br>**Superfamily:** Fe-only hydrogenase<br>**Family:** Fe-only hydrogenase<br>**PDB entry:** PDBe RCSB PDBj
98	c3lx4B_	Alignment	not modelled	19.3	57	**PDB header:**oxidoreductase<br>**Chain:** B: **PDB Molecule:**fe-hydrogenase;<br>**PDBTitle:** stepwise [fefe]-hydrogenase h-cluster assembly revealed in the2 structure of hyda(deltaefg)<br>**PDB Entry:** PDBe RCSB PDBj
99	d1hfel1	Alignment	not modelled	11.0	57	**Fold:** Fe-only hydrogenase<br>**Superfamily:** Fe-only hydrogenase<br>**Family:** Fe-only hydrogenase<br>**PDB entry:** PDBe RCSB PDBj
